# Comparison of motor skill learning, grip strength and memory recall on land and in chest-deep water

**DOI:** 10.1371/journal.pone.0202284

**Published:** 2018-08-14

**Authors:** Eadric Bressel, Michael N. Vakula, Youngwook Kim, David A. E. Bolton, Christopher J. Dakin

**Affiliations:** 1 Department of Kinesiology and Health Science, Utah State University, Logan, Utah, United States of America; 2 Movement Research Clinic, Sorenson Legacy Foundation Center for Clinical Excellence, Utah State University, Logan, Utah, United States of America; 3 Sport Performance Research Institute, Auckland University of Technology, Auckland, New Zealand; University of Illinois at Urbana-Champaign, UNITED STATES

## Abstract

Immersion in chest-deep water may augment explicit memory in healthy adults however, there is limited information on how this environment might affect implicit memory or motor learning. The purpose of this study was to compare the speed and accuracy for learning a motor skill on land and in chest-deep water. Verbal word recall and grip strength were included to gain a more complete understanding of the intervention. Sixty-two younger adults (age = 23.3 ± 3.59 yrs.) were randomly assigned to either a water group immersed to the xiphoid or a land group. Participants in both groups completed the same eight practice trials of a mirror-drawing task on two separate days. Outcome measures for this task included time and error numbers to complete each drawing. The number of words recalled using a 12 word recall test, and peak grip strength using a hand dynamometer were measured each day of testing. The influence of environment and repeated practice on each outcome measure were assessed with an analysis of variance and effect sizes (ES). Time and errors for both groups significantly decreased with practice (p < 0.01, ES = 0.11–0.28), however the drawing time was greater in water than on land for trials 1, 5, and 6 (ES = 0.50–0.55). There was a 7% increase in words recalled (9.24 ± 1.19 vs 8.60 ± 1.19) and a 16% increase in grip strength (405 ± 104 vs 342 ± 83) for water than land groups (ES 0.54–0.64). Healthy adults in chest-deep water and on land display comparable mirror-drawing speed and accuracy after minimal practice. Curiously, water immersion may augment verbal word recall and grip strength abilities.

## Introduction

A primary goal of physical rehabilitation is to restore function in a timely manner using evidence-based treatments. Indeed, how quickly a treatment restores function is restricted by biological-related factors (e.g., type of tissue injured), patient-related factors (e.g., age), and treatment-related factors (e.g., type of treatment received). Regarding the latter, multiple evidence-based treatments may resolve the impairment, yet the rate of improvement following each treatment intervention may differ drastically. For instance, stroke survivors often display reduced gait velocity compared to controls [1] and task-specific high velocity gait training appears to improve gait velocity more quickly compared to conventional gait training and therapy [2]. Considering that many stroke survivors often do not receive enough therapy to improve some activities of daily living to the level of independence [3], it is imperative for clinicians to use treatments that minimize the time-course to recovery.

We have recently observed that gait training in chest-deep thermoneutral water significantly improved measures of gait [4] and mobility [5] in patients with osteoarthritis after one-week of training with no improvements after equivalent land training. We have also observed that healthy younger and older adults tended to make fewer ‘cognitive’ errors on an explicit auditory memory task while immersed chest-deep in water than on land during single and dual-task conditions [6, 7]. Other researchers have reported greater blood flow velocity in the cerebral arteries [8, 9] and greater oxygenated hemoglobin concentrations in sensory and motor areas of the cerebral cortex [10] during partial water immersion compared to land. Collectively, these studies indicate that the aquatic environment may augment cognitive and motor processes, but it is unclear whether the augmented cognitive and motor processes observed during partial water immersion influence the speed of recovering motor function.

Recovery of motor function is fundamentally a process of relearning motor skills and is dependent on intensive practice [11]. By repeating complex motor skills over and over again (practice), the skills are refined and through procedural learning, stored as implicit memory. This motor skill refinement and maintenance is essential for independent involvement in the activities of daily living. Often, a mirror-drawing task is used to asses implicit memory performance [12]. The task requires a person to trace a shape, and stay within the boundaries of a double line, while observing an inverted reflection of their hand through a mirror. Motor learning for the task is often assessed using improvements in tracing time (e.g., speed) and accuracy (e.g., fewer errors) over repeated trials and through the assessment of bilateral transfer [13]. Of importance here, the mirror-drawing task can be performed in chest-deep water and the speed and accuracy of learning may be observed within a few practice sessions [14].

The aim of the current study was to compare the speed and accuracy of learning by evaluating mirror-drawing skill on land and in chest-deep water. Evaluating motor skill learning or implicit memory in the aquatic environment is an original aspect of this study. We expected the time-course or rate of learning to be faster and with fewer errors in water than on land. We also assessed verbal memory recall (i.e., explicit memory) and grip strength to better understand how the environment more generally affected cognitive and motor function. Previously, we observed improvements in auditory memory while immersed in chest-deep water than on land [6, 7], and in the current study we extend this observation to a verbal memory task. Additionally, by including the verbal memory task, the study was better positioned to evaluate if partial water immersion has a similar or differential effect on explicit and implicit learning. Results of this study will address the following research question: Does partial water immersion influence motor skill learning, verbal memory recall, and grip strength? This research may help maximize the benefits of physical rehabilitation.

## Materials and methods

This study utilized a single-blind between-group research design. A treatment group practiced the mirror-drawing task in chest-deep water and a control group practiced the mirror-drawing task on land. The study was designed to optimize the consolidation of learning by using a two-day training session. Since another hallmark of learning is how well a motor skill can transfer, we also included a bilateral transfer assessment in the research design. To minimize experimental bias, the participants were blinded to the group assignment.

### Participants

**Fig 1:**
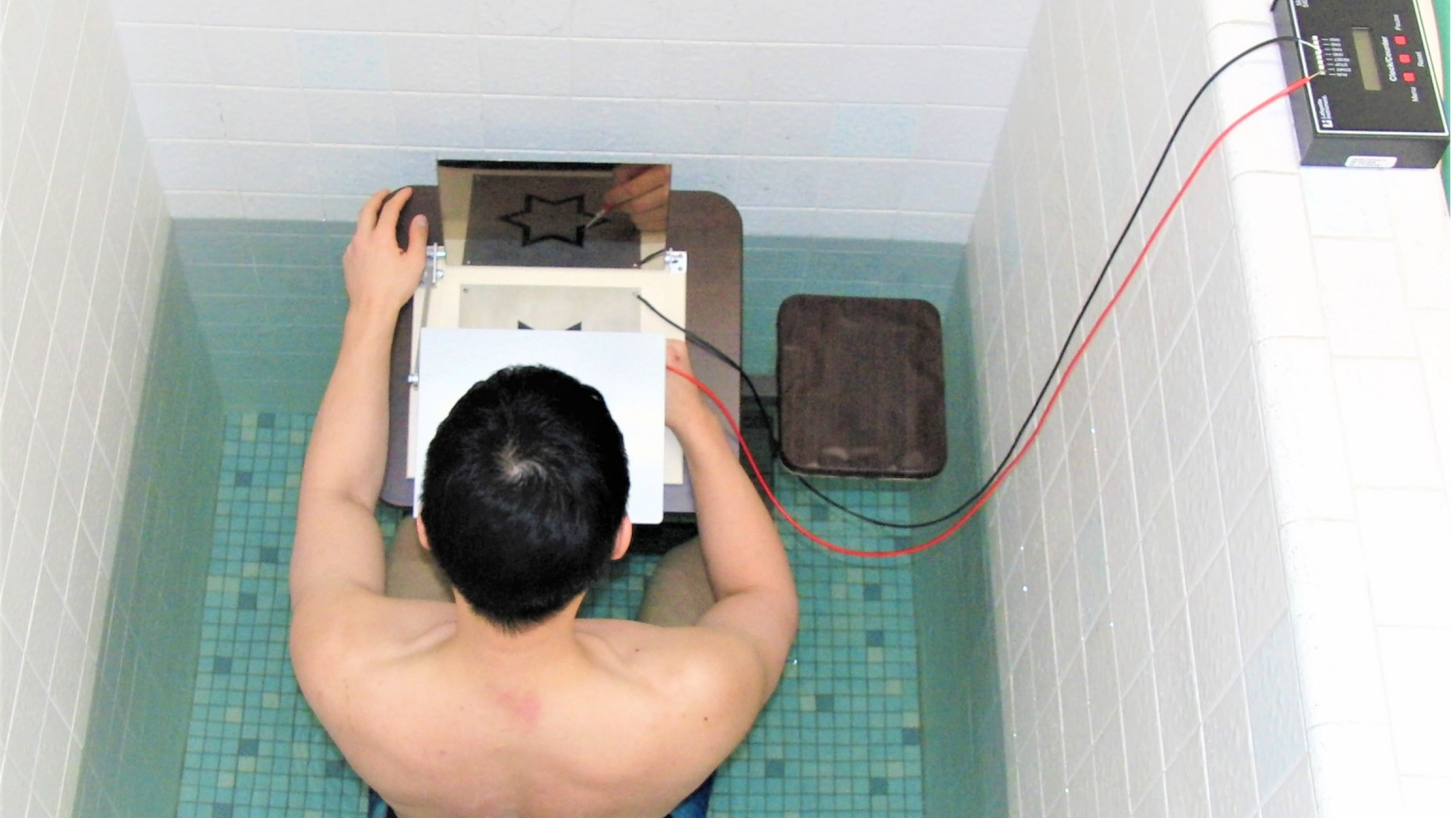
Mirror-drawing task.

A total of 64 healthy adults between the ages of 18–40 yrs. were asked to participate in the study. Participants were recruited from a university setting via word-of-mouth referrals. Volunteering participants were excluded if they had previously performed a mirror-drawing task or self-reported any visual or motor disorders that would prevent them from tracing a star shape on a flat surface while seated. The participants were assigned to either the treatment (water) or control (land) group using a pseudo random technique. The participants’ physical characteristics and their self-reported activity level, educational level, and handedness are reported in Table 1. The two groups did not differ significantly (p > 0.05) on any measure reported in Table 1 based on independent t-Tests or chi-square for the categorical data. The sample size was based on effect sizes (ES) computed from previous studies using a similar mirror-drawing task paradigm [12]. Participants were required to sign an informed consent form. This study and the informed consent form were approved by the university Institutional Review Board. Additionally, the individual in Fig 1 has given written informed consent (as outlined in PLOS consent form) to publish these case details.

**Table 1:** Participant characteristics (Mean, ±SD) for the water (n = 31) and land (n = 31) groups.

Characteristic	Water	Land	p value
Age (yrs)	23.8 (3.80)	22.8 (3.37)	p = 0.26
Mass (kg)	72.1 (14.7)	73.3 (15.6)	p = 0.75
Height (m)	1.75 (0.09)	1.73 (0.09)	p = 0.68
Handedness	1.10 (0.30)	1.16 (0.37)	p = 0.46
Gender	3.39 (0.50)	3.52 (0.51)	p = 0.32
Activity level (days/week)	4.20 (1.86)	4.80 (1.82)	p = 0.10
Level of education (1–5)	4.00 (1.00)	4.00 (1.00)	p = 0.61
Heart rate (bpm)	60.8 (9.37)	62.1 (10.6)	p = 0.63

Handedness; 1 = right hand, 2 = left hand.

Level of education 1 = grade school; 2 = jr high; 3 = high school; 4 = college; 5 = graduate school.

Gender; 3 = male; 4 = female.

Note: Mode and range reported for level of education.

### Procedures

Participants in each group attended two training sessions separated by 24 hrs. The duration of each training session was about 30 min with the first 10 min of the first training session being devoted to collecting participant physical characteristics. The location of each training session took place in the same quiet room that contained a hydrostatic weighing tank (Fig 1). The room was climate-controlled with the air and water temperature regulated to 24.5 ± 0.49° C and 30.8 ± 1.40° C, respectively. This water temperature was chosen as it is consistent with temperatures reported in previous research that focused on aquatics and cognition [6–9]. The temperature also appears to be thermoneutral as it does not change skin temperature during immersion [9]. The location of the training, the training itself, and the procedures for testing were the same for the treatment and control groups. The only difference being the treatment group training and testing occurred while being immersed in water to the depth of the inferior aspect of the xiphoid process. This depth was adjusted for each person and was chosen because it is consistent with previous aquatic-based studies [6] and was a depth that permitted mirror-drawing on a table in a seated position without having the arms immersed in water (Fig 1).

#### Mirror-drawing task

Participants were seated in front of an auto scoring mirror tracer (Model 58024E; Lafayette Instrument, Lafayette, IN. USA) that was mounted to a table angled to 15° (Fig 1). Participants were first given instructions on the goal of the task and to adjust the occluder to maximize vision of the hand and star in the mirror-inverted image. After 5 min of remaining in the seated position either on land or in chest-deep water, participants were given the following instructions: “Place the stylus down at any point on the star, between the two borderlines. Without lifting the stylus up, trace completely around the star, as quickly as possible without sacrificing accuracy. Accuracy will be defined as your ability to stay within the lines.” It was emphasized that tracing errors and time were equally important to successful completion of the task. The number of errors (moving outside the boundary) to complete each tracing was automatically recorded and the time to complete each tracing was recorded with a standard stop watch.

On each day of training, participants were asked to complete two blocks of four trials each using their dominant hand, with a 10 s inter-trial interval and 10 min inter-block interval. Two training blocks of four practice trials was chosen as it appears to be sufficient practice to improve mirror-drawing performance on land [14]. Immediately before training on the first day and after training on the second day, participants completed a preliminary tracing test with their dominant and non-dominant hand in random order for the assessment of transfer.

#### Verbal word recall task

At the completion of the second block of training on the first day, participants were asked to memorize a list of 12 words using the Memory Assessment Scale methods [15]. Participants were then asked to recall words from the list in any order on the first day (short-delay recall) and after the second block of training on the second day (long-delay recall). Participants were given the following instructions when asked to memorize the list of words on the first day: *“I’m going to read a list of 12 words to you*. *When I’m finished*, *I want you to tell me as many words as you can remember*. *It doesn’t matter in what order you say them*. *We will practice the list six times or until you remember all 12 words*. *Do you understand*? *Listen carefully*. *Here are the words (1 word/sec)*: *Blue*, *England*, *Sparrow*, *Yellow*, *Italy*, *Paris*, *Crow*, *Orange*, *Denver*, *Japan*, *Athens*, *Robin*. *Now tell me as many of the words as you can remember”*. For the short-delay recall assessment on the second day of testing the following instructions were provided: “*Remember that list of words that you learned the last time we met*? *Tell me as many of those words as you can remember*. *Begin*.” A smartphone was used to record the verbal responses and then the replay function was used to score the performance on a standardized scoring template [15]. The outcome measure of interest for this test was the number of words recalled at each time point.

#### Hand grip strength

Hand grip strength was measured during the 10 min inter-block interval on the first and second day of training using standardized methods [16]. Participants were seated in their respective environments (i.e., land or water) with their elbow fully extended and arm resting on the table. Participants were asked to grip the hand dynamometer (Plus Digital Hand Dynamometer; JLW Instruments, Chicago, IL. USA) and squeeze as hard as possible for 5 s while they received encouragement. The test was repeated three times for each hand, which was randomly assigned for the first test. For the remaining 10 min inter-block interval, heart rate from a finger pulse oximeter (SportStat; Nonin Medical, Inc., Minneapolis, MN. USA) was recorded each minute for descriptive purposes.

#### Data analysis

Consistent with previous research, the time (s) required to complete each trial and the number of errors per trial served as dependent measures for the mirror-drawing task [12, 14]. The rate of improvement from trial to trial was assessed by computing a time change score for each trial pair across both days of testing using the following equation where T = time to completion: %T_1-2_ = [(T_1_-T_2_) x 100]/T_1_. This computation of improvement was performed sequentially for each trial (%T_1-2,_ %T_2-3_%T_3-4_,…%T_15-16_) and was comparable with improvement computations used in previous mirror-drawing literature [14].

Regarding the verbal word recall test, the number of words recalled from the first trial on day one and two were used for subsequent statistical analyses. The peak grip strength value (kg) among the three trials for each hand were recorded and converted to Newtons (N) for subsequent statistical analyses.

#### Statistical analyses

Pre-analysis screening was performed for each dependent measure to test for normality and homogeneity of variance using visual inspection of the histograms, Shapiro-Wilk scores, and skewness. Data that violated these assumptions were log transformed and retested. For presentation of the results, the data that were log transformed were transformed back.

Dependent measures for the mirror-drawing task (i.e., time, errors, & improvement) were analyzed using a 2 (environment) x 16 (trial) repeated measures analysis of variance (ANOVA) with environment (land vs water) as an independent factor. If significant main effects were observed for the trial factor, multiple comparisons between sequential trial pairs collapsed across environment were performed using a Bonferroni correction. If significant interactions were observed, follow-up independent t-Tests were performed on the environment factor to determine where differences occurred across trials. Transfer of learning was assessed for the time measure using a 2 (environment) x 2 (pretest vs posttest for non-dominant hand) ANOVA. Verbal word recall comparisons were assessed using a 2 (environment) x 2 (immediate vs delayed recall) ANOVA and grip strength comparisons were assessed using a 2 (environment) x 2 (handedness) x 2 (day 1 vs day 2) ANOVA. The alpha level was set to 0.05 for all comparisons and any violations of sphericity for repeated measures were corrected with a Greenhouse-Geisser adjusted F. To appreciate the meaningfulness of any statistical differences, Cohen’s d effect sizes (ES’s) were computed and interpreted using the following scale: 0.0–0.2 = small, 0.2–0.5 = moderate, and > 0.5 = large ES. Finally, the average heart rate for each environment was computed for descriptive purposes in Table 1.

## Results

Sixty-four participants were screened for inclusion in the study and two participants were excluded because of previous mirror-drawing experience. Sixty-two participants were included in the study and assessed as planned. Statistical test assumptions were met for mirror-drawing and verbal word recall measures after log transformation, whereas grip strength measures did not require log transformation to meet test assumptions.

### Mirror-drawing task

**Fig 2:**
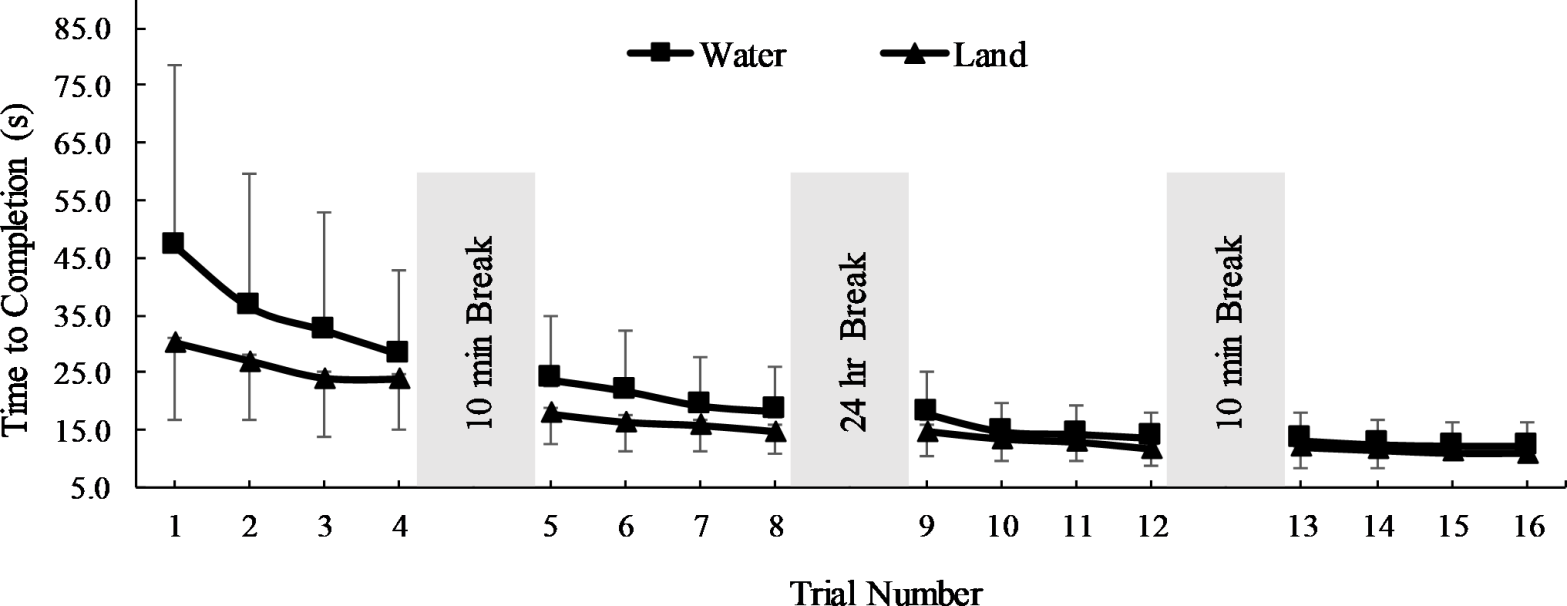
Time to complete the star tracing (mean, SD).

The ANOVA for the time measure revealed no main effect for the environment factor (F = 2.96, p = 0.09), a significant main effect for the trial factor (F = 196, p = 0.001), and a significant environment by trial interaction (F = 2.64, p = 0.04). Follow-up multiple comparisons for the trial factor revealed the time to complete each star tracing significantly decreased over all trial sequences between 1–12 (p < 0.01, ES = 0.11–0.28) except between trials 3–4 (p = 0.19) and 8–9 (p = 0.74, Fig 2). The interaction revealed that, although time to completion decreased for land and water groups, the completion time was greater in water than on land for trials 1, 5, and 6 (p < 0.03, ES = 0.50–0.55).

**Fig 3:**
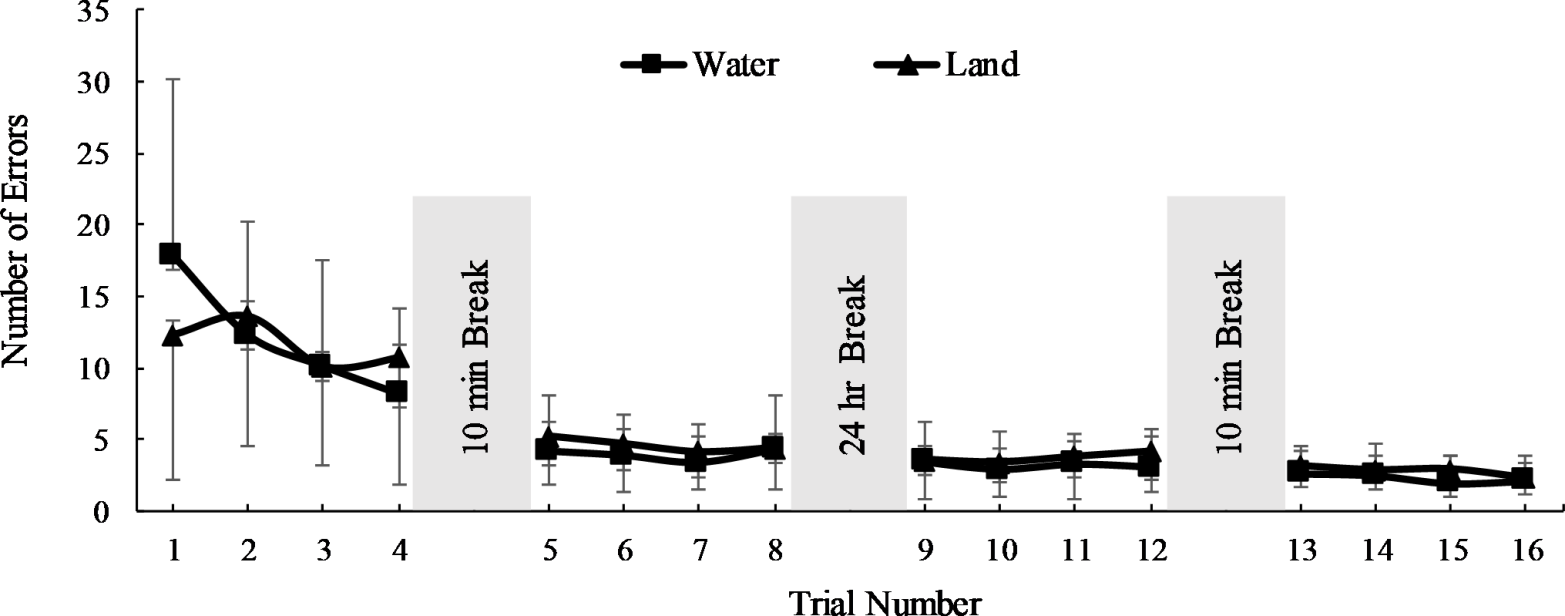
Number of errors to complete the star tracing (mean, SD).

The same analysis on the number of errors revealed no main effect for the environment factor (F = 1.00, p = 0.32) and a significant main effect for the trial factor (F = 45.0, p = 0.001). However, an environment by trial interaction was not observed (F = 1.42, p = 0.18). Follow-up comparisons for the trial factor revealed the number of errors were only lower between trials 2–3 (p = 0.05, ES = 0.31) and 4–5 (p = 0.001, ES = 0.65, Fig 3). Individual error numbers and time to completion for each participant that completed the mirror-drawing task are reported in S1 Table.

Unlike the other mirror-drawing measures, the improvement scores displayed a significant main effect for the environment factor (F = 5.91, p = 0.02) and the trial factor (F = 3.44, p = 0.005), yet an environment by trial interaction was not observed (F = 1.17, p = 0.32). Multiple comparisons revealed improvement scores increased and decreased across some trial sequences (p < 0.002, ES = 0.46–0.65), however, values were 32% greater overall for water than land environments (ES = 0.67, Table 2).

**Table 2:** Mean (SD) improvement in time to complete the star tracing task across trials on land and in water.

Sequence	Water	Land	Marginal mean
%T_1-2_	17.8 (19.9)	6.42 (22.8)	12.1 (22.0)
%T_2-3_	5.26 (32.3)	7.26 (34.6)	6.26 (33.2)
%T_3-4_	6.29 (26.4)	-4.86 (32.9)	0.72 (30.1)
%T_4-5_	12.0 (17.4)	21.3 (17.8)	16.7 (18.0)^b^
%T_5-6_	7.43 (11.9)	6.84 (13.7)	7.14 (12.7)^b^
%T_6-7_	9.50 (13.5)	2.30 (12.7)	5.90 (13.5)
%T_7-8_	3.31 (16.0)	5.26 (15.8)	4.28 (15.8)
%T_8-9_	0.81 (16.3)	-3.28 (25.5)	-1.23 (21.3)
%T_9-10_	14.4 (12.8)	8.03 (10.8)	11.2 (12.2)^b^
%T_10-11_	2.83 (9.27)	3.48 (7.75)	3.15 (8.48)^b^
%T_11-12_	3.66 (10.3)	8.24 (12.0)	5.95 (11.3)
%T_12-13_	24.2 (11.9)	17.8 (11.5)	21.0 (12.1)
%T_13-14_	3.55 (13.7)	3.25 (9.62)	3.40 (11.8)
%T_14-15_	0.24 (14.3)	2.82 (14.9)	1.53 (14.5)
%T_15-16_	0.52 (8.58)	-2.11 (24.7)	0.79 (18.4)
Group mean	6.08 (2.95)^a^	4.09 (2.95)	

^a^Group mean different from land condition (p < 0.02)

^b^Marginal mean different from previous trail (p < 0.003).

**Fig 4:**
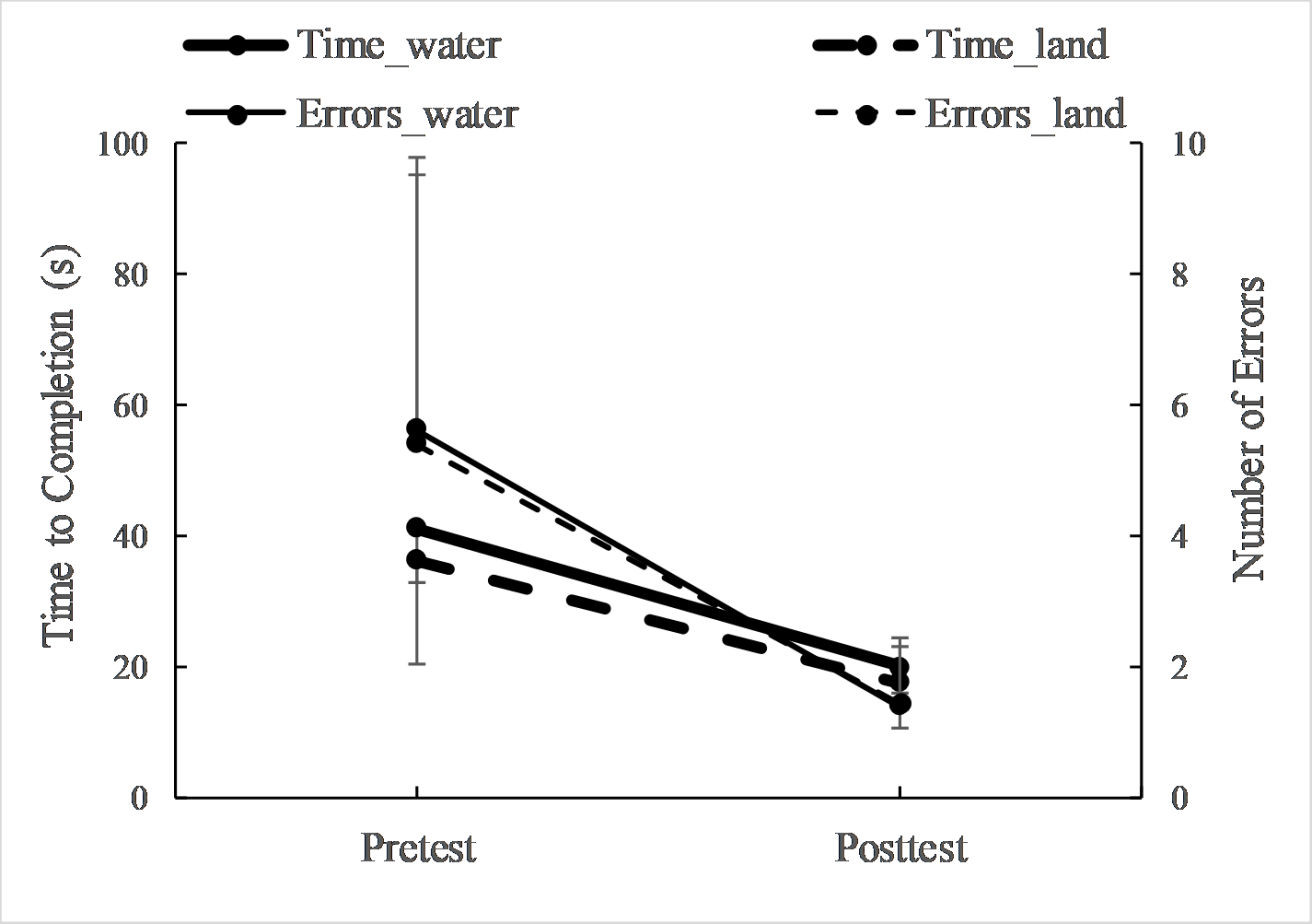
Time and errors to complete the star tracing (mean, SD) using the non-dominant hand before (pretest) and after training (posttest).

The assessment of transfer to the non-dominant hand revealed that time to completion and errors were more than 60% lower for the posttest versus pretest, regardless of environment (F = 177–237, p < 0.001, ES > 1.13, Fig 4). Additionally, there was no main effect for the environment factor (p = 0.15–0.80).

### Verbal word recall task

The number of words recalled were 35% greater for the delayed versus immediate recall, regardless of environment (F = 112, p < 0.001, ES > 1.64). The environment factor was significant (F = 5.70, p < 0.001) with a 7% increase in words recalled for water than land environments (ES = 0.54, Table 3).

**Table 3:** Mean (SD) number of words recalled immediately and one day later (delayed) on land and in water.

	Water	Land	Marginal mean
Immediate	7.93 (1.14)	7.35 (1.87)	7.64 (1.56)
Delayed	10.5 (0.93)	9.84 (1.75)	10.2 (1.44)^b^
Group mean	9.24 (1.19)^a^	8.60 (1.19)	

^a^Group mean different from land condition (p < 0.02)

^b^Marginal mean different from immediate recall (p < 0.001).

### Hand grip strength

**Fig 5:**
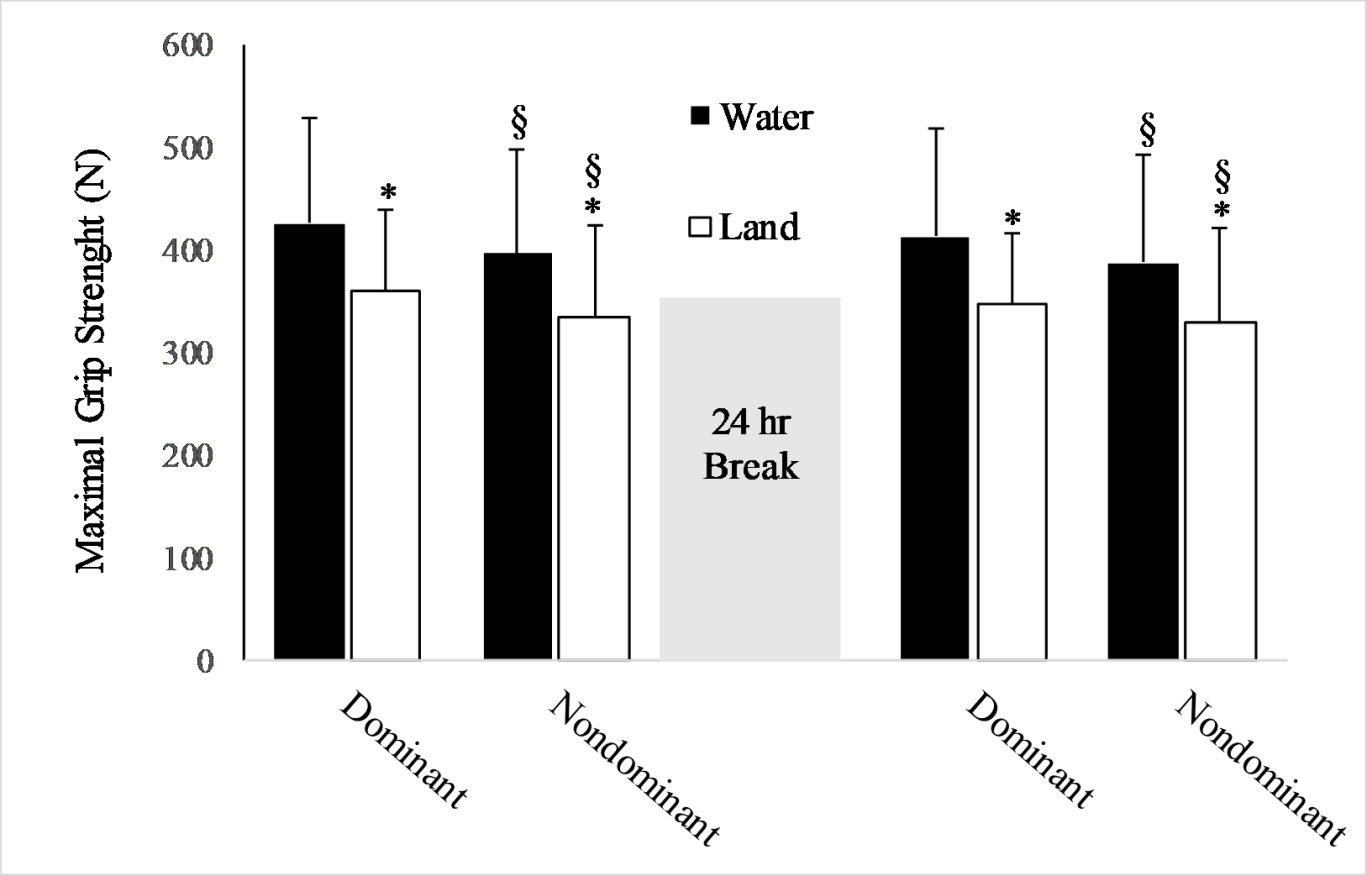
Peak grip strength (mean, SD) for the dominant and nondominant hand. *Significantly different from water. §Significantly different from dominant hand; p < 0.05.

Regarding grip strength, a significant main effect for the environment (F = 7.48, p = 0.01) and handedness (F = 29.1, p = 0.001) was observed. The day of testing was not significant (F = 7.21, p = 0.06) and there was no significant interactions (p > 0.48). Grip strength values in water were 16% greater than on land (ES = 0.64), and as expected, peak values were 6% lower for the non-dominant than dominant hand (ES = 0.32; Fig 5).

## Discussion

To our knowledge, this is the first study to observe the effects of partial water immersion on procedural learning or implicit memory. We hypothesized the rate of improvement for learning would be greater and with fewer errors in water than on land. The results of the current study support the former and reject the later aspect of this hypothesis. That is, rate of improvement was greater overall for the water group (Table 2), yet errors were not different between groups (Fig 3).

The speed and accuracy data in the current study are in accord with previous mirror-drawing literature using similar methods. To be clear, no previous studies, that the authors are aware of, have used the mirror-drawing task in an aquatic environment. Relative to previous studies using the mirror-drawing task, Rodrigue et al. [17] observed that healthy younger adults on land displayed a first trial time to completion in about 45 s, which is consistent with values in the current study for the land group (e.g., 47.2 ± 30.2 s; Fig 2). With respect to accuracy, Rodrigue and co-workers reported about eight errors for a healthy younger-aged group and in the current study 12.4 ± 10.1 errors were observed for the land group during the first trial (Fig 3). After 16 trials, Rodrigue et al. reported about one error and in the current study 2.32 ± 1.70 errors were observed for the same group comparison. It is important to note that subtle differences in methods (e.g., star size, participant age, error detection methods) may influence the uniformity of speed and accuracy between mirror-drawing studies.

What is not clear from the data is why the time to complete the initial practice trials took longer in the water group. Given that the physical characteristics of the groups were similar (Table 1) and the methods were similar between groups, the differences in time were likely related to the environment. For instance, the novel properties of water (e.g., buoyance) may have produced a less stable base of support or an attentional focus shift or “interference” due to competing demands for neural resources [18]. Evidence of interference is typically revealed as a decrease in task performance during a dual task activity (e.g., walking + talking) [19]. The greater initial time to completion observed in the current study may be evidence of instability or interference for the aquatic group (e.g., water immersion + mirror-drawing). This conjecture will indeed need to be tested formally in future research.

Although the focus of the current study was on motor skill learning using the mirror-drawing task, we included an explicit verbal memory recall test to better understand how the aquatic environment influenced cognitive function. Previously, we observed that younger and older adults tended to make fewer errors on an explicit auditory memory task while immersed chest-deep in water than on land [6, 7]. The results of the current study extend this observation to a verbal memory recall task, as evidenced in Table 3. The differences in words recalled between groups was only about 7%, and the ES was 0.54 suggesting moderate clinical relevance It may be expected that an older population with greater variability from the highest possible score will display larger ES’s, as evidenced in recent work [7]. The mechanism for improved explicit memory in water is unknown, but as proposed previously [6, 7], may be related to a change in parasympathetic drive or greater cerebral blood flow. For instance, hydrostatic pressure applied to a person in chest-deep thermoneutral water produces a chain of events: Peripheral blood volume shifts to the thoracic region, central blood volume and stroke volume increase, baroreflexes are stimulated, and vagal tone and parasympathetic drive are stimulated. Curiously, this hemodynamic event during water immersion does not appear to influence ones’ implicit memory or their ability to learn the mirror drawing task.

Collectively, the cognitive results of the current study indicate that partial water immersion may have a differential effect on explicit and implicit learning. That is, partial water immersion may influence accuracy on a memory recall task but not a mirror drawing motor skill task. The observation that memory recall and motor skill learning are dissociated is not a new concept, as reported in 1962 by Milner et al. with patient H.M. [20]. Perhaps the more relevant observation is that after only six short practice trials, both groups behaved the same with respect to time, errors, and percent improvement on the motor skill task. The clinical relevance of this observation is that younger adults may not experience any improvements or deteriorations in overall speed or accuracy of learning a motor skill in chest-deep water. They will however gain some improvements in verbal word recall.

Regarding the consolidation of motor skill learning, it may be observed in Figs 2 and 3 that regardless of the environment, time and errors between the last trial of the first day and the first trial of the second day were not different suggesting the aquatic environment did not positively or negatively influence the consolidation of learning. Further, both groups displayed a significant and meaningful improvement in motor skill learning for the non-dominant hand (Fig 4) suggesting the aquatic environment did not influence the transfer of learning. Combined with the other classical markers of skill learning (i.e. increased performance, less variability, and retention) these results indicate that participants in a water and land environment ultimately learn just as well.

Perhaps the most unexpected result in the current study was that grip strength was greater for the water than land group, regardless of the day tested (Fig 5). The differences between groups were meaningful based on the large ES (e.g., 0.64). Measures of muscle strength are generally viewed as indicators of the integrity of nervous system [21], and although the nature of the relationship is unclear, grip strength is positively associated with cognitive decline on explicit memory tests [22, 23]. Many aquatic-based exercise studies examine how resistance training in water influences strength measures on land. The results of the current study suggest the capacity to improve grip strength in the water may be greater than on land, yet this conjecture will need to be more formally tested.

There are limitations of the current study. For example, the immediate effects of aquatic immersion and not the longitudinal effects were examined. It is reasonable to expect that chronic exposure to the aquatic environment may normalize mirror-drawing, verbal word recall and grip strength values as participants become more accustomed to the novel stimuli of the aquatic environment. Additionally, only healthy younger adults were tested. It might be expected that individuals with mild cognitive impairment or dementia will more likely display implicit or explicit memory errors because attentional resources may be compromised. Finally, a baseline strength measure was not included and only one measure of strength was assessed in the current study. It can not be assumed that baseline grip strength was similar between groups and that lower limb strength was different between groups. Future research initiatives include the need to test other domains of cognitive function (e.g., executive function) and its relationship with cerebral activity to elucidate possible mechanisms of association. Additional measures of strength may also be appropriate to better understand how the aquatic environment might augment or diminish motor function when compared to the same activities performed on land.

### Conclusions

Motor skill learning was ultimately not different when training in water than on land. This was evident using standard markers of motor improvement including: (1) Performance improvement in completing the task, (2) more consistent performance, (3) retention, and (4) transfer of learning. These findings are valuable given that it suggests all of the benefits of learning motor skills can be attained within a safe, low-impact aquatic environment. Consistent with previous research, explicit memory was augmented and curiously, grip strength was also augmented in water compared to land groups, which suggests there may be unexplored benefits with this form of therapy.

## Supporting information

S1 Table Error numbers and time to completion for each participant that completed the mirror-drawing task. (XLSX)

## References

[1] Goldie PA, Matyas TA, Evans OM. Deficit and change in gait velocity during rehabilitation after stroke. Arch Phys Med Rehabil. 1996;77(10):1074–82. 8857890 10.1016/s0003-9993(96)90072-6

[2] Pohl M, Mehrholz J, Ritschel C, Ruckriem S. Speed-dependent treadmill training in ambulatory hemiparetic stroke patients: a randomized controlled trial. Stroke. 2002;33(2):553–8. 11823669 10.1161/hs0202.102365

[3] Kimberley TJ, Samargia S, Moore LG, Shakya JK, Lang CE. Comparison of amounts and types of practice during rehabilitation for traumatic brain injury and stroke. J Rehabil Res Dev. 2010;47(9):851–62. 21174250 10.1682/jrrd.2010.02.0019

[4] Roper JA, Bressel E, Tillman MD. Acute aquatic treadmill exercise improves gait and pain in people with knee osteoarthritis. Arch Phys Med Rehabil. 2013;94(3):419–25. 10.1016/j.apmr.2012.10.027 23131526

[5] Denning WM, Bressel E, Dolny DG. Underwater Treadmill Exercise as a Potential Treatment for Adults With Osteoarthritis. International Journal of Aquatic Research and Education. 2010;4:70–80.

[6] Schaefer SY, Louder TJ, Foster S, Bressel E. Effect of Water Immersion on Dual-task Performance: Implications for Aquatic Therapy. Physiother Res Int. 2016;21(3):147–54. 10.1002/pri.1628 25891889

[7] Bressel E, Louder TJ, Raikes AC, Alphonsa S, Kyvelidou A. Water Immersion Affects Episodic Memory and Postural Control in Healthy Older Adults. J Geriatr Phys Ther. 2018; Forthcoming. 10.1519/JPT.0000000000000192 29738406

[8] Carter HH, Spence AL, Pugh CJ, Ainslie P, Naylor LH, Green DJ. Cardiovascular responses to water immersion in humans: impact on cerebral perfusion. Am J Physiol Regul Integr Comp Physiol. 2014;306(9):R636–40. 10.1152/ajpregu.00516.2013 24553298 PMC4010659

[9] Pugh CJ, Sprung VS, Ono K, Spence AL, Thijssen DH, Carter HH, et al The effect of water immersion during exercise on cerebral blood flow. Med Sci Sports Exerc. 2015;47(2):299–306. 10.1249/MSS.0000000000000422 24977699

[10] Sato D, Onishi H, Yamashiro K, Iwabe T, Shimoyama Y, Maruyama A. Water immersion to the femur level affects cerebral cortical activity in humans: functional near-infrared spectroscopy study. Brain Topogr. 2012;25(2):220–7. 10.1007/s10548-011-0204-z 22193361

[11] Winstein CJ, Stewart JC. Conditions of task practice for individuals with neurological impairments Textbook of Neural Repair and Neurorehabilitation Medical Neurorehabilitation. Cambridge: Cambridge University Press; 2006 p. 89–102.

[12] Julius MS, Adi-Japha E. A Developmental Perspective in Learning the Mirror-Drawing Task. Front Hum Neurosci. 2016;10:83 10.3389/fnhum.2016.00083 26973498 PMC4773595

[13] Latash ML. Mirror Writing: Learning, Transfer, and Implications for Internal Inverse Models. J Mot Behav. 1999;31(2):107–11. 10.1080/00222899909600981 11177624

[14] Magallon S, Narbona J, Crespo-Eguilaz N. Acquisition of Motor and Cognitive Skills through Repetition in Typically Developing Children. PLoS One. 2016;11(7):e0158684 10.1371/journal.pone.0158684 27384671 PMC4934913

[15] Williams JM. Memory assessment scales professional manual Psychological Assessment Resources, Inc Odessa, Florida 1991 p. 1–127.

[16] Roberts HC, Denison HJ, Martin HJ, Patel HP, Syddall H, Cooper C, et al A review of the measurement of grip strength in clinical and epidemiological studies: towards a standardised approach. Age Ageing. 2011;40(4):423–9. 10.1093/ageing/afr051 21624928

[17] Rodrigue KM, Kennedy KM, Raz N. Aging and longitudinal change in perceptual-motor skill acquisition in healthy adults. J Gerontol B Psychol Sci Soc Sci. 2005;60(4):P174–81. 15980284 10.1093/geronb/60.4.p174

[18] Passingham RE. Attention to action. Philos Trans R Soc Lond B Biol Sci. 1996;351(1346):1473–9. 10.1098/rstb.1996.0132 8941959

[19] Woollacott M, Shumway-Cook A. Attention and the control of posture and gait: a review of an emerging area of research. Gait Posture. 2002;16(1):1–14. 12127181 10.1016/s0966-6362(01)00156-4

[20] Milner B, Squire LR, Kandel ER. Cognitive neuroscience and the study of memory. Neuron. 1998;20(3):445–68. 9539121 10.1016/s0896-6273(00)80987-3

[21] Christensen H, Mackinnon AJ, Korten A, Jorm AF. The "common cause hypothesis" of cognitive aging: evidence for not only a common factor but also specific associations of age with vision and grip strength in a cross-sectional analysis. Psychol Aging. 2001;16(4):588–99. 11766914 10.1037//0882-7974.16.4.588

[22] Alfaro-Acha A, Al Snih S, Raji MA, Kuo YF, Markides KS, Ottenbacher KJ. Handgrip strength and cognitive decline in older Mexican Americans. J Gerontol A Biol Sci Med Sci. 2006;61(8):859–65. 16912105 10.1093/gerona/61.8.859 PMC1635471

[23] Taekema DG, Ling CH, Kurrle SE, Cameron ID, Meskers CG, Blauw GJ, et al Temporal relationship between handgrip strength and cognitive performance in oldest old people. Age Ageing. 2012;41(4):506–12. 10.1093/ageing/afs013 22374646

